# Predicted impingement-free motion amplitudes in reverse total shoulder arthroplasty differs between supine computed tomography and standing biplanar x-ray imaging: a pilot study

**DOI:** 10.1016/j.xrrt.2025.05.022

**Published:** 2025-06-14

**Authors:** Florent Moissenet, Sandrine Bousigues, Sana Boudabbous, Davide Cabral, Laurent Gajny, Nicola Hagemeister, Nicolas Holzer

**Affiliations:** a Kinesiology Laboratory, Geneva University Hospitals and University of Geneva, Geneva, Switzerland; b Biomechanics Laboratory, Geneva University Hospitals and University of Geneva, Geneva, Switzerland; c Laboratoire de Biomécanique et Mécanique des Chocs, Université Gustave Eiffel and Université Claude Bernard Lyon 1, Lyon, France; d Department of Radiology, Geneva University Hospitals, Geneva, Switzerland; e Arts et Métiers Institute of Technology, Institut de Biomécanique Humaine Georges Charpak, Paris, France; f Ecole de Technologie Supérieure, Montréal, Canada; g Laboratoire de recherche en imagerie et orthopédie, Centre de recherche du Centre hospitalier de l’Université de Montréal, Montréal, Canada; h Orthopaedic Surgery and Musculoskeletal Trauma Care Division, Department of Surgery, Geneva University Hospitals, Geneva, Switzerland

**Keywords:** Biplanar radiography, Scapulothoracic joint, Surgical planning, Joint biomechanics, Shoulder arthroplasty, Personalised medicine

Preoperative planning software is widely used to assist surgeons in selecting and positioning implant components for reverse total shoulder arthroplasty (rTSA).^14^ These tools rely on patient-specific bone surface models derived from preoperative supine computed tomography (CT) scans and predict postoperative impingement-free motion amplitudes by simulating uniplanar humeral movements. However, a major limitation is that the CT scans are acquired in the supine position, which fails to account for the scapula posture in a standing position—a factor critical for clinical assessments.^11^ This discrepancy can significantly influence surgical planning.^9^^,^^11^ Studies by Moroder et al^10^ and Kriechling et al^6^ emphasize the importance of considering the scapula posture, as postoperative observations show its substantial impact on functional outcomes.

Despite growing recognition of its importance, standardized and reliable methods for measuring scapula posture in the standing position are still lacking. Current studies, including those by Moroder et al^10^ and Kriechling et al,^6^ often rely on supine CT scans for adjusting the scapula posture. However, Matsumura et al^8^ demonstrated that the scapula posture significantly differs between supine and standing positions, raising concerns about the clinical relevance of supine imaging, which is influenced by the positioning of the shoulder and upper limb during recording. Moreover, defining the scapula posture in a thorax- or patient-referenced coordinate system (CS) typically requires whole thoraco-abdominal CT scans, which increases radiation exposure.^9^^,^^11^

To address these limitations, several alternative measurement methods are available. In particular, a novel solution by Bousigues et al^2^ involves low-dose biplanar radiography in a standing posture. When combined with conventional supine CT-based bone surface models, this approach could provide a clinically relevant tool for determining the scapula posture in the standing position prior to motion amplitude predictions. The aim of this pilot study was to evaluate the feasibility of this approach in determining impingement-free motion amplitudes. We hypothesized that adjusting the patient-specific scapula posture would significantly impact both the predicted impingement-free motion amplitudes (eg, flexion), range of motion (ROM, eg, flexion-extension), and the sites of impingement.

## Methods

### Participants

This monocentric retrospective study was approved by the Cantonal Research Ethics Commission of Geneva (CER 2019-00069). Preoperative CT and biplanar x-ray images were retrieved from patients undergoing elective rTSA at Geneva University Hospitals between September and December 2023. All participants provided written informed consent.

### Medical imaging

**Figure 1:**
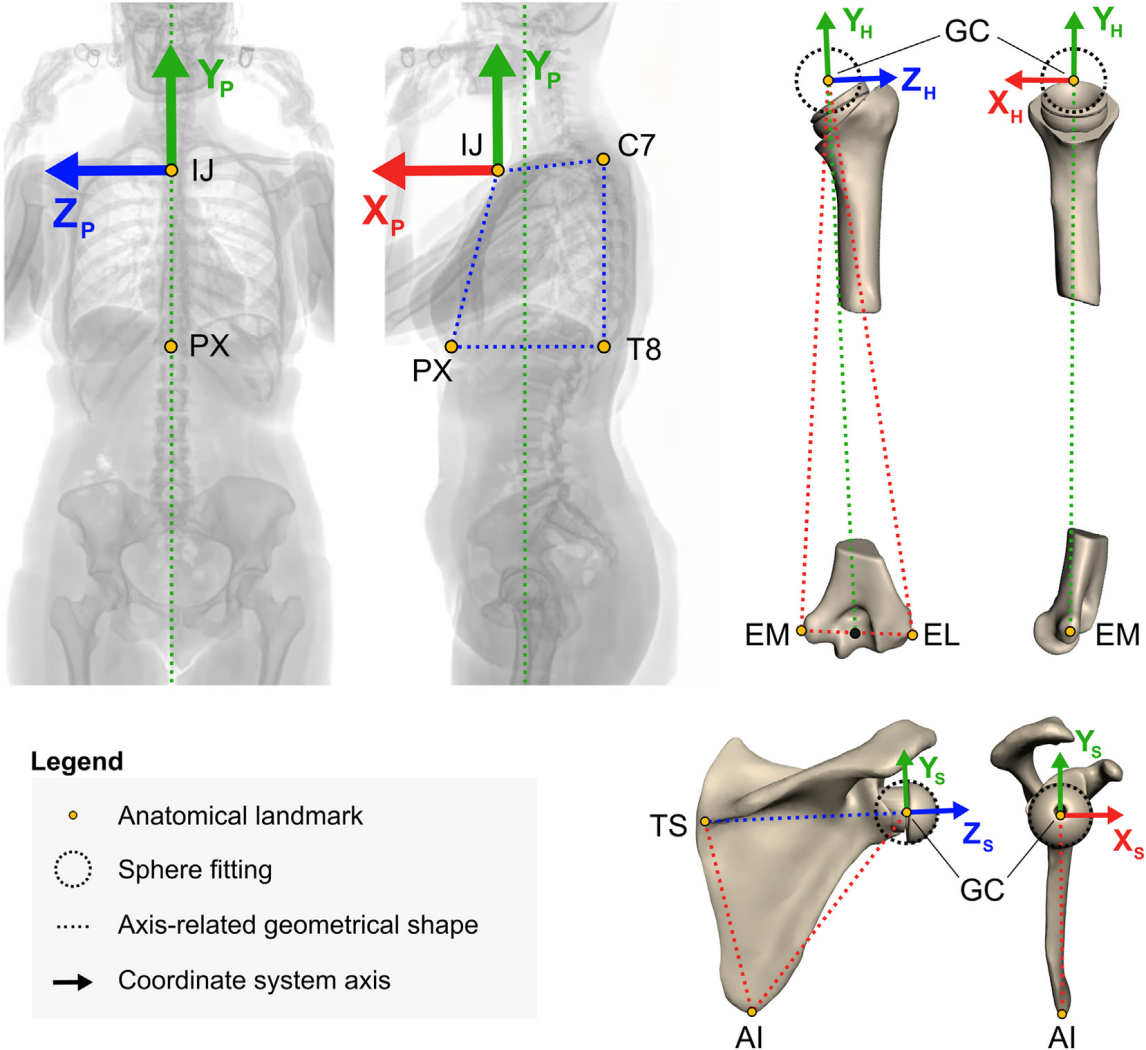
Coordinate system used to simulate humerus motions. The patient-referenced CS was defined as follows: $O_{P}$ is centered on the deepest point of IJ, $Y_{P}$ represents the vertical axis of the biplanar x-ray images, pointing superiorly, $Z_{P}^{*}$ is normal to a plane passing through the processus spinosus of the seventh cervical vertebra (C7), the processus spinosus of the eighth thoracic vertebra (T8), IJ, and the PX, pointing right, $X_{P}$ is determined as the cross-product of $Y_{P}$ and $Z_{P}^{*}$, and $Z_{P}$ is determined as the cross-product of $X_{P}$ and $Y_{P}$, pointing anteriorly. Thorax landmarks were identified by sphere fitting on skin fiducials in IdefX. The scapula local CS was defined as follows: $O_{S}$ is the origin centered at the GC, $X_{S}$ is orthogonal to a plane defined by the TS, the AI, and GC, pointing anteriorly, $Z_{S}$ lies on a line crossing TS and GC, pointing laterally, and $Y_{S}$ is determined as the cross-product of $Z_{S}$ and $X_{S}$, pointing superiorly. The humerus local CS was defined as follows^16^: $O_{H}$ is the origin centered at GC, $X_{H}$ is orthogonal to a plane defined by the most caudal point on lateral EL, the most caudal point on medial EM, and GC, pointing anteriorly, $Y_{H}$ lies on a line crossing GC and the middle of EL and EM, pointing superiorly, and $Z_{H}$ is determined as the cross-product of $X_{H}$ and $Y_{H}$, pointing laterally. All anatomical landmarks were identified in Slicer 3D (5.2.2, https://www.slicer.org/).^4^*CS*, coordinate system; *IJ*, incisura jugularis; *PX*, processus xiphoideus; *GC*, glenosphere center; *TS*, trigonum spinae; *AI*, angulus inferior; *EL*, humeral epicondyle; *EM*, humeral epicondyle; *3D*, 3-dimensional.

CT images were acquired in the supine position following our standard rTSA protocol using a 24-row CT unit (Somatom Drive; Siemens Healthcare, Erlangen, Germany). Biplanar x-ray images were taken in 40° axial rotation relative to the anterior-posterior view (as illustrated in Fig. 2 in Bousigues et al, 2023,^2^ and reproduced in the supplementary materials), with patients standing and arms relaxed, using the EOS acquisition system (ATEC Spine, Carlsbad, CA, USA). Skin fiducials were placed on anatomical landmarks of the thorax (Fig. 1) to assist in identification. This view was selected to avoid bony superimposition of both shoulders and the spine.^2^ The observed patient-specific scapula posture was expressed as an internal-external rotation, upward-downward rotation, and anterior-posterior tilt (see section *Prediction of Postoperative Impingement-Free Motion Amplitudes*). Based on the internal rotation (IR) of the scapula, patients were categorized according to Moroder et al^9^ into three types: type A (IR ≤ 36°), type B (36° < IR < 47°), and type C (IR ≥ 47°). Additional biplanar x-ray images were taken in the coronal and sagittal planes to measure the sacral slope orientation, which allowed for categorizing patients based on the Roussouly classification, a system that describes sagittal spinal alignment.^13^ For all imaging, patient positioning was strictly controlled, and the staff followed a posture checklist to minimize discrepancies.

### Preoperative planning

Patient-specific surface bone models of the scapula and humerus were segmented using a semiautomatic method (Mimics; Materialise, Leuven Belgium). A shoulder surgeon performed virtual implantations using a planning software (MyShoulder; Medacta Intl., Castel San Pietro, Switzerland) as part of our standard 3-dimensional (3D) planning process. Each patient received a correction for the native glenoid orientation using autologous bone grafting to achieve a baseplate neutral version and inferior inclination of −5°. The planning surgeon was blinded to the scapula posture type, as done in the study by Moroder et al.^11^ For each patient, the implant positioning remained unchanged throughout the study.

### 2-dimensional–3-dimensional registration

The scapula surface model and biplanar x-ray images were imported into image-processing software (IdefX, Institut de Biomécanique Humaine Georges Charpak, Arts et Métiers Sciences et Technologies, Paris, France; LIO, École de technologie supérieure, Montreal, Québec, Canada) for 2-dimensional (2D)–3D registration. A single operator performed a manual registration, adjusting the model's position and orientation to align with the radiological contours in both x-ray views, focusing on the glenoid, coracoid process, scapular spine, and medial border.

### Prediction of postoperative impingement-free motion amplitudes

**Figure 2:**
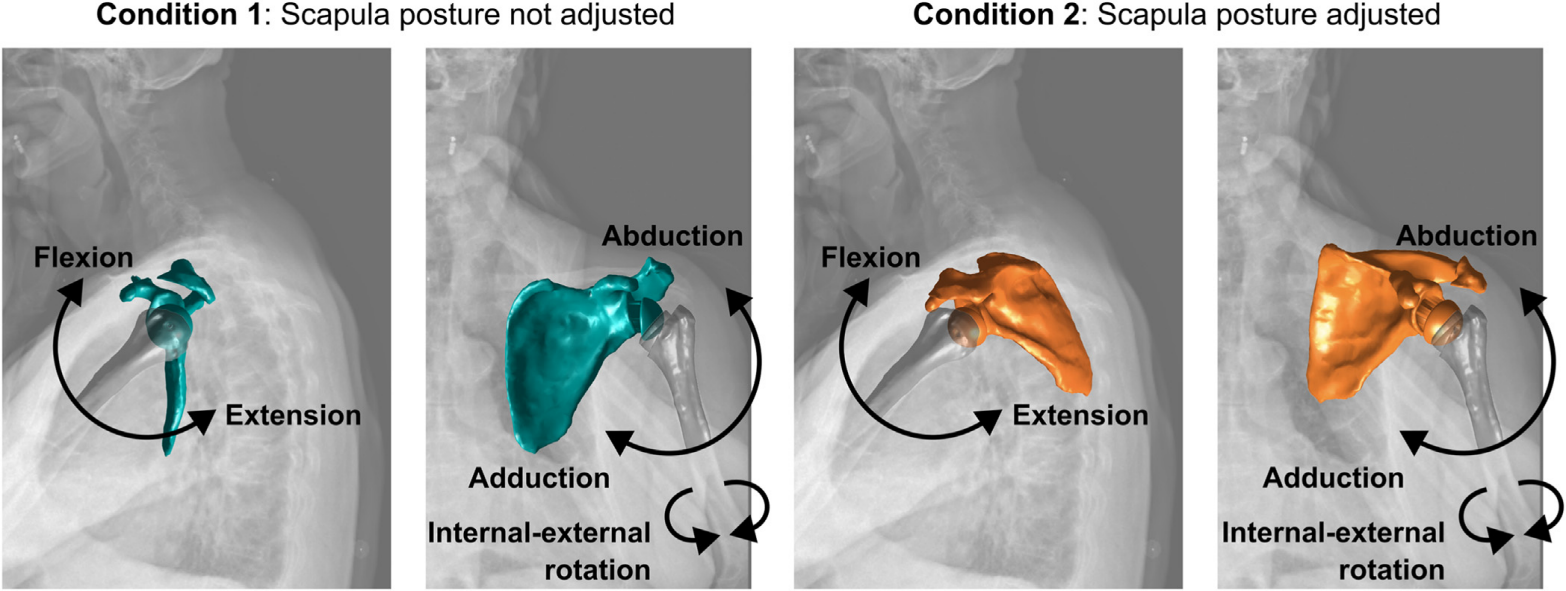
Illustration of the impingement-free motion amplitude simulations highlighting the impact of the scapula posture adjustment on the predictions.

To assess the effect of the scapula posture on the predicted impingement-free motion amplitudes and related ROM, two simulation conditions were compared (Fig. 2). The first condition was performed without adjusting the scapula posture. It used the scapula local CS to simulate humeral motions relative to the scapular planes. The second condition was performed with a patient-specific adjustment of the scapula posture. It used the patient-referenced CS to simulate humeral motions relative to the body planes. The CSs are defined in Figure 1. Simulations were performed in MATLAB (R2022b; MathWorks, Natick, MA, USA) by imposing humeral elevations of 0°-180° in the sagittal (flexion and extension) and coronal (abduction and adduction) planes, with humeral rotations of 0°, ±30°, and ±60°, and internal–external rotations (at 0° of abduction), using a YX'Y″ Euler sequence^16^ (step size: 1°). The patient-specific scapula posture was expressed in the patient-referenced CS and computed using a YX'Z″ Euler sequence.^16^ The sites of impingement were detected by calculating the shortest Euclidean distance between the humerus and scapula surface model vertices (using the *pdist2* function in MATLAB). A conflict was defined as a distance of less than 0.5 mm, excluding contact points between the humerus and glenoid implants, and focusing on non–implant-related impingements.

### Statistical analyses

The primary parameters analyzed were the elevation angle at which the first bony conflict occurred and the related site of impingement (the sites of impingement are defined in Fig. 4). Median values and interquartile ranges summarized the predicted postoperative impingement-free amplitude of each motion (ie, flexion, extension, abduction, adduction, internal, and external rotation) and the ROM in each plane (ie, flexion–extension, abduction–adduction, internal–external rotation), for each condition. The effect of the scapula posture was assessed using a Friedman test (paired samples) across all simulated motions and humeral rotations. Post hoc Wilcoxon signed rank tests were performed to compare conditions for each humeral rotation.

**Figure 3:**
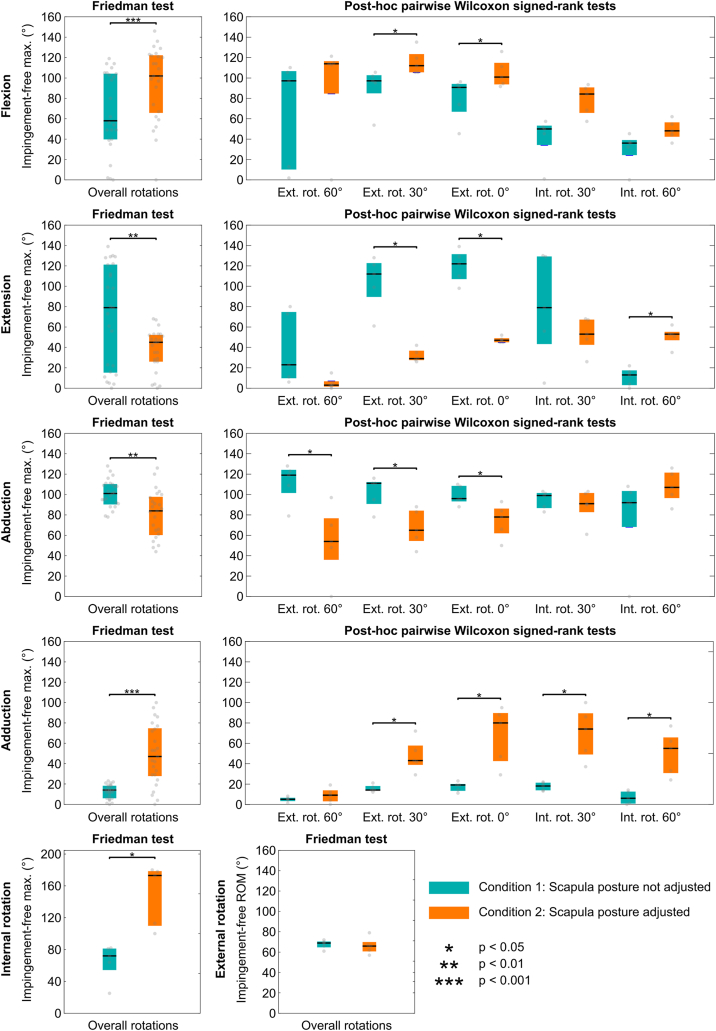
Boxplots illustrating the effect of the scapula posture adjustment on postoperative impingement-free motion amplitudes. The colored box spans from the first to the third quartile, with the *black line* representing the median, and the *gray dots* the values.

**Figure 4:**
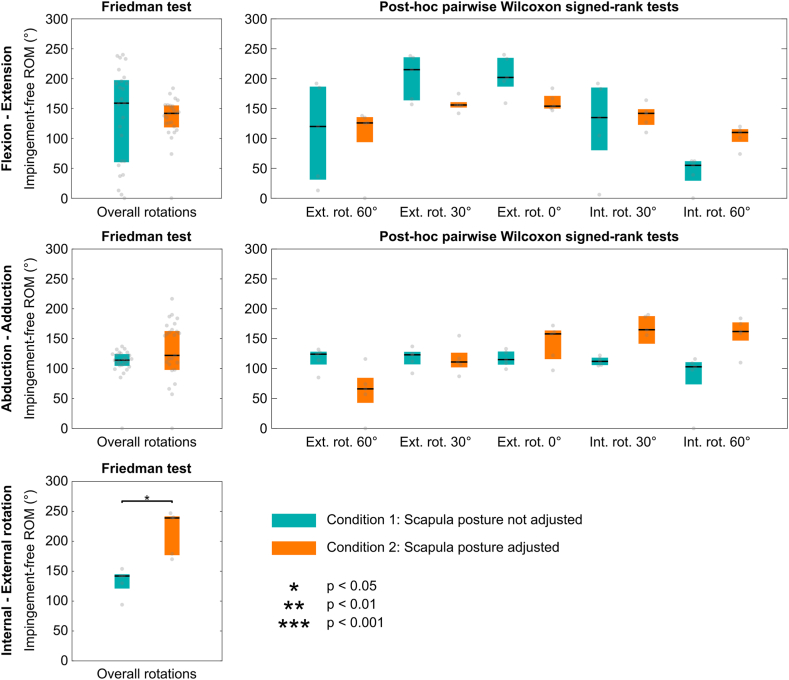
Boxplots illustrating the effect of the scapula posture adjustment on postoperative impingement-free ranges of motion. The colored box spans from the first to the third quartile, with the *black line* representing the median, and the *gray dots* the values.

## Results

### Participants

Five patients (3 women, 168.2 ± 9.7 cm, 84.0 ± 20.5 kg) were included in this study. Both type 1 (short lumbar lordosis and long thoracic kyphosis) and type 3 (normal spine curves) were observed according to the Roussouly classification.^13^ Types A, B, and C from the Moroder classification^9^ were observed. Further epidemiological details are provided in Table I.

**Table I:** Characteristics of the participants and virtual implants used in the planning process.

Patient						Posture		Humeral implants		Glenoid implants		
ID	Age	Gender	Height (cm)	Body mass (kg)	Side	Roussouly classif.	Moroder classif.	Inclination (°)	Retrotorsion (°)	Inclination (°)	Retroversion (°)	Glenosphere size (mm)
P01	74	Male	175	100	Left	Type 3	Type B (38°)	135	20	−2	0	39
P02	70	Male	169	105	Right	Type 1	Type C (49°)	135	20	−5	0	39
P03	58	Male	180	82	Left	Type 3	Type B (43°)	135	20	−5	0	39
P04	78	Female	160	80	Right	Type 1	Type A (28°)	135	20	−5	0	36
P05	77	Female	157	53	Right	Type 3	Type C (51°)	135	20	−5	5	36

### Predicted postoperative impingement-free motion amplitudes

Predicted postoperative impingement-free motion amplitudes and ROM are presented as boxplots in Figures 3 and 4, respectively, for each condition (with and without scapula posture adjustment). Detailed outcomes (values for each patient, descriptive and inferential statistics) are reported in the supplementary materials. Adjustments in scapular posture produced significant changes in impingement-free motion amplitudes. During flexion, the parameter increased significantly by 44° (*P* < .001), while extension was accompanied by a significant decrease of 34° (*P* < .001). Similarly, abduction decreased by 15° (*P* < .001), contrasted by a 33° significant increase during adduction (*P* < .001). These differences were further influenced by the humeral rotation applied during the elevation motions. Regarding rotations, only the IR showed a significant increase of 101° (*P* < .05). However, only the internal–external rotation ROM increasing significantly by 97° (*P* < .05).

### Predicted sites of impingement

**Figure 5:**
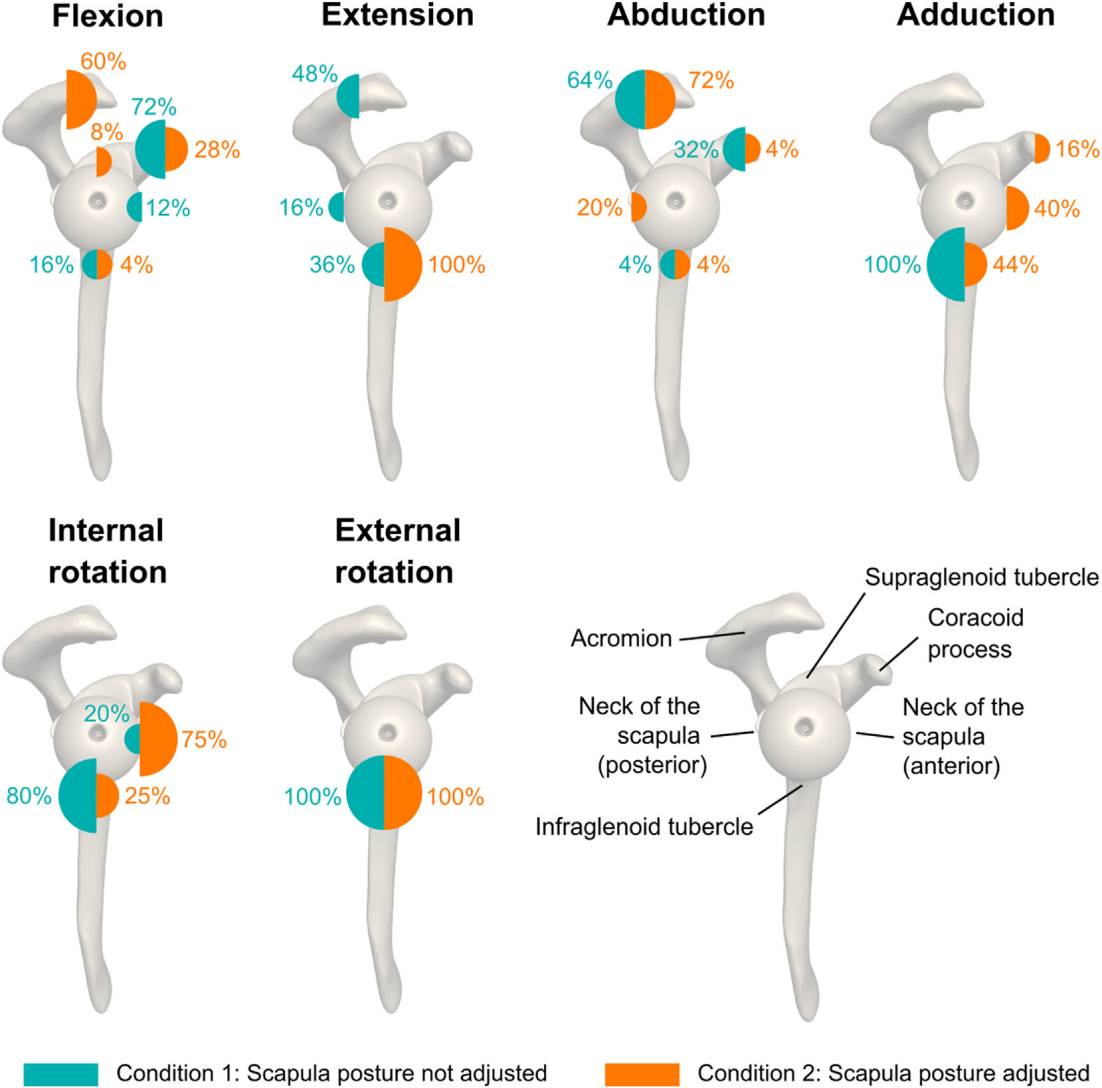
Map of the sites of impingement and related occurrences across scapula posture conditions and humerus rotations.

Predicted sites of impingement are shown in Figure 5 for each condition (with and without scapula posture adjustment). The scapula posture adjustment led to changes in the location of the bony conflicts, independent of humeral rotation. During flexion, the site of impingement shifted posteriorly with scapula posture adjustment, involving the acromion, coracoid process, supraglenoid tubercle, and infraglenoid tubercle. Without scapula posture adjustment, impingement was observed at the coracoid process, anterior neck of the scapula, and infraglenoid tubercle. During extension, impingement occurred exclusively at the infraglenoid tubercle when the scapula posture was adjusted. Without scapula posture adjustment, impingement was observed at multiple locations, including the acromion, posterior neck of the scapula, and infraglenoid tubercle. During abduction, the site of impingement remained largely unchanged, with primary conflicts at the acromion, coracoid process, and infraglenoid tubercle. However, with scapula posture adjustment, impingement also occurred at the posterior neck of the scapula. During adduction, the site of impingement shifted anteriorly with scapula posture adjustment, involving the coracoid process, anterior neck of the scapula, and infraglenoid tubercle. Without scapula posture adjustment, impingement was only observed at the infraglenoid tubercle. During internal and external rotation, the site of impingement remained largely unchanged, with primary conflicts at the anterior neck of the scapula (external rotation) and infraglenoid tubercle (internal and external rotation).

## Discussion

The aim of this pilot study was to demonstrate the applicability of a novel biplanar radiographic approach for adjusting scapula posture in preoperative planning for rTSA. Our preliminary results confirmed the importance of considering the scapula posture in rTSA planning, highlighting its significant impact on predicted impingement-free motion amplitudes, ROM, and sites of impingement, regardless of the patient's posture as assessed by the Roussouly and Moroder classifications (Video clip 1).

### Scapula posture adjustment approach

Building on the work of Bousigues et al,^2^ the proposed approach captures the scapula posture in the standing position using two orthogonal x-ray images. This method improves the 2D–3D registration accuracy compared to single-image approaches.^12^^,^^15^ By utilizing a low–radiation dose system, the approach also allows for whole-body imaging, which not only considers the thorax posture but also the spine and overall posture of the patient. This method addresses key challenges in assessing the scapula posture compared to the current practices and aligns with the clinical recommendations from Moroder et al.^9^, ^10^, ^11^

The approach consists of three key steps: first, obtaining standing x-ray images, which is essential until standing CT scans become more common^8^; second, establishing a patient-referenced CS, bypassing the International Society of Biomechanics (ISB) recommendations for defining the thorax CS,^16^ which may be influenced by postural changes such as kyphosis; and third, performing a 2D–3D registration. While this registration process is currently manual, it could benefit from semiautomated solutions in the future.^17^ These steps are straightforward, align with existing practices, and could significantly enhance the preoperative rTSA planning, thereby improving surgical precision and patient outcomes.

### Predicted postoperative impingement-free motion amplitudes

The predicted impingement-free motion amplitudes without adjusting the scapula posture are consistent with findings from recent literature. For flexion at 0° external rotation, the amplitudes in the literature span from 83.1 ± 14.6° to 127 ± 27°,^1^^,^^3^^,^^5^^,^^18^ compared to 98° [72°-102°] (median [interquartile range]) in our study. For extension at 0° external rotation, Lädermann et al^7^ reported 81.3 ± 28.1°, which is lower than our values of 122° [107°-132°]. For abduction at 0° external rotation, reported amplitudes range from 77 ± 13° to 120 ± 30°,^1^^,^^3^^,^^5^^,^^7^^,^^18^ compared to 96° [93°-109°] in this study. For adduction at 0° external rotation, Lädermann et al^7^ reported 28.6 ± 10.7°, which is slightly higher than our values of 19° [13°-20°]. Only results for external rotation at 0° abduction were found in the literature, and reported amplitudes span from 15 ± 21° to 50 ± 19°,^1^^,^^3^^,^^5^ compared to 69° [65°-71°] in this study. These differences in reported amplitudes can likely be attributed to variations in implants, 3D planning software, and surgical techniques. Notably, bone graft lateralization was planned in our study to maximize impingement-free motion amplitudes.

The impact of the scapula posture adjustment on impingement-free motion amplitudes also aligns with previous studies. Moroder et al^11^ showed that the scapular IR, downward rotation, and anterior tilt correlate with decreased abduction and extension amplitudes when comparing type A, B, and C patients. We observed similar trends when comparing nonadjusted and adjusted scapula postures. However, unlike Moroder et al,^11^ who observed no significant changes in flexion, we identified a pronounced increase in flexion amplitude. This discrepancy may be attributable to methodological differences or disparities in sample size (n = 5 vs. n = 30).

Interestingly, we found no significant alterations in overall flexion–extension or abduction–adduction ROM. This suggests that the loss of extension (or abduction) from adjusting the scapula posture is offset by a gain in flexion (or adduction), thereby shifting the neutral alignment in each plane. In contrast, a significant increase in the internal–external rotation range was observed. This finding aligns with Moroder et al,^9^ who reported that scapular IR (increasing from type A to type C patients) is linked to higher IR amplitudes across different scapula posture types.

### Predicted sites of impingement

The predicted impingement sites without adjusting the scapula posture align with prior findings. Specifically, Lädermann et al^7^ identified the coracoid process as the main site of impingement in flexion, the acromion and infraglenoid tubercle in extension, the acromion in abduction, and the infraglenoid tubercle in both adduction and external rotation.

To our knowledge, this is the first study to examine the effect of the scapula posture adjustments on the predicted sites of impingement. We observed significant differences in these locations between conditions, emphasizing the potential importance of the scapula posture in refining preoperative assessments. However, further research is needed to assess the clinical impact of these adjustments, particularly once implant settings are updated.

### Limitations

This study provides valuable insights into the impact of the scapula posture on rTSA planning, but there are several limitations. First, the small sample size (n = 5) limits the generalizability of our findings. However, this pilot study was intended to establish the feasibility of the proposed approach. Larger-scale studies are needed to confirm these results and assess variability across different patient populations.

Second, the manual 2D–3D registration process introduces potential bias or variability, which may affect the reproducibility and reliability of the results. This manual procedure has not yet been formally validated. Future research should focus on validating automated or semiautomated registration processes to improve consistency and enhance clinical feasibility.

Finally, this study did not establish direct correlations between predicted impingement-free motion amplitudes and clinical outcomes. Future studies should aim to validate the clinical relevance of these findings by assessing postoperative function and pain outcomes.

## Conclusion

This pilot study demonstrates the feasibility and importance of incorporating the scapula posture into the preoperative planning for rTSA using a low-dose biplanar radiographic approach. Our findings reveal significant effects on predicted impingement-free motion amplitudes and sites of impingement, highlighting the potential of this method to refine surgical precision and improve patient outcomes. Future studies should focus on validating these results in larger cohorts, automating key processes, and correlating predicted changes with clinical outcomes to maximize the translational impact of this approach.

## Acknowledgment

This project was made possible thanks to the LIA-EVASYM shared laboratory.

## Disclaimers:

Funding: The authors did not receive any financial remuneration for thy study.

Conflicts of interest: The authors, their immediate families, and any research foundation with which they are affiliated have not received any financial payments or other benefits from any commercial entity related to the subject of this article.

## Supplementary data

Supplementary Material 3

Video 1 Study context and main outcomes.
