# Supplementary material for: Predicted impingement-free motion amplitudes in reverse total shoulder arthroplasty differs between supine computed tomography and standing biplanar x-ray imaging: a pilot study
Source: JSES Rev Rep Tech. 2025 Jun 14;5(4):1050–7. doi: 10.1016/j.xrrt.2025.05.022 (PMC12573451; doi:10.1016/j.xrrt.2025.05.022)
Supplement: Supplementary Material 1 [file mmc1.docx]

**Predicted impingement-free motion amplitudes in reverse total shoulder arthroplasty differs between supine CT and standing biplanar X-ray imaging: a pilot study**

Florent Moissenet^1,2^, Sandrine Bousigues^3^, Sana Boudabbous^4^, Davide Cabral^4^, Laurent Gajny^5^, Nicola Hagemeister^6,7^, Nicolas Holzer^2,8^

*^1^ Kinesiology Laboratory, Geneva University Hospitals and University of Geneva, Geneva, Switzerland*

*^2^ Biomechanics Laboratory, Geneva University Hospitals and University of Geneva, Geneva, Switzerland*

*^3^ Laboratoire de Biomécanique et Mécanique des Chocs, Université Gustave Eiffel and Université Claude Bernard Lyon 1, Lyon, France*

*^4^ Department of Radiology, Geneva University Hospitals, Geneva, Switzerland*

*^5^ Arts et Métiers Institute of Technology, Institut de Biomécanique Humaine Georges Charpak, Paris, France*

*^6^ Ecole de Technologie Supérieure, Montréal, Canada*

*^7^ Laboratoire de recherche en imagerie et orthopédie, Centre de recherche du Centre hospitalier de l’Université de Montréal, Montréal, Canada*

*^8^ Orthopaedic Surgery and Musculoskeletal Trauma Care Division, Department of Surgery, Geneva University Hospitals, Geneva, Switzerland*

**Supplementary material 1**

(see next page)

Table S1 - Detailed descriptive and inferential statistics for impingement-free motion amplitudes

| Motion | Condition | Median  (°) | IQR  (°) | Friedman test | Humeral rotation | Condition | Median (°) | IQR  (°) | Wilcoxon test |
| --- | --- | --- | --- | --- | --- | --- | --- | --- | --- |
| Flexion | 1 | 58 | [40 104] | χ^2^ (df) = 21.54 (1)  p = 3.454e^-6^ | Ext. rot. 60° | 1 | 105 | [11 115] | p = 0.3452 |
|  |  |  |  |  |  | 2 | 123 | [92 126] |  |
|  |  |  |  |  | Ext. rot. 30° | 1 | 105 | [92 111] | p = 0.0431 |
|  |  |  |  |  |  | 2 | 121 | [114 133] |  |
|  |  |  |  |  | Ext. rot.  0° | 1 | 98 | [72 102] | p = 0.0431 |
|  | 2 | 102 | [66 122] |  |  | 2 | 109 | [101 124] |  |
|  |  |  |  |  | Int. rot.  30° | 1 | 54 | [37 58] | p = 0.0679 |
|  |  |  |  |  |  | 2 | 91 | [71 98] |  |
|  |  |  |  |  | Int. rot.  60° | 1 | 39 | [26 42] | p = 0.0796 |
|  |  |  |  |  |  | 2 | 52 | [46 61] |  |
| Extension | 1 | 79 | [15 121] | χ^2^ (df) = 7.10 (1)  p = 0.0077 | Ext. rot. 60° | 1 | 23 | [10 75] | p = 0.1380 |
|  |  |  |  |  |  | 2 | 3 | [2 7] |  |
|  |  |  |  |  | Ext. rot. 30° | 1 | 112 | [90 123] | p = 0.0431 |
|  |  |  |  |  |  | 2 | 29 | [28 37] |  |
|  |  |  |  |  | Ext. rot.  0° | 1 | 122 | [107 132] | p = 0.0431 |
|  | 2 | 45 | [26 52] |  |  | 2 | 47 | [45 49] |  |
|  |  |  |  |  | Int. rot.  30° | 1 | 79 | [43 129] | p = 0.3452 |
|  |  |  |  |  |  | 2 | 53 | [43 67] |  |
|  |  |  |  |  | Int. rot.  60° | 1 | 13 | [3 18] | p = 0.0431 |
|  |  |  |  |  |  | 2 | 53 | [47 55] |  |
| Abduction | 1 | 101 | [90 110] | χ^2^ (df) = 7.10 (1)  p = 0.0076 | Ext. rot. 60° | 1 | 119 | [102 124] | p = 0.0431 |
|  |  |  |  |  |  | 2 | 54 | [36 77] |  |
|  |  |  |  |  | Ext. rot. 30° | 1 | 111 | [91 112] | p = 0.0431 |
|  |  |  |  |  |  | 2 | 65 | [54 84] |  |
|  |  |  |  |  | Ext. rot.  0° | 1 | 96 | [93 109] | p = 0.0431 |
|  | 2 | 84 | [60 98] |  |  | 2 | 78 | [62 86] |  |
|  |  |  |  |  | Int. rot.  30° | 1 | 99 | [87 102] | p = 0.7150 |
|  |  |  |  |  |  | 2 | 91 | [83 102] |  |
|  |  |  |  |  | Int. rot.  60° | 1 | 92 | [68 104] | p = 0.0796 |
|  |  |  |  |  |  | 2 | 107 | [97 122] |  |
| Adduction | 1 | 14 | [6 18] | χ^2^ (df) = 25.54 (1)  p = 4.329e^-7^ | Ext. rot. 60° | 1 | 5 | [3 7] | p = 0.2785 |
|  |  |  |  |  |  | 2 | 9 | [3 14] |  |
|  |  |  |  |  | Ext. rot. 30° | 1 | 14 | [14 18] | p = 0.0431 |
|  |  |  |  |  |  | 2 | 43 | [39 58] |  |
|  |  |  |  |  | Ext. rot.  0° | 1 | 19 | [13 20] | p = 0.0431 |
|  | 2 | 47 | [28 75] |  |  | 2 | 80 | [43 90] |  |
|  |  |  |  |  | Int. rot.  30° | 1 | 18 | [14 21] | p = 0.0431 |
|  |  |  |  |  |  | 2 | 74 | [49 90] |  |
|  |  |  |  |  | Int. rot.  60° | 1 | 6 | [1 13] | p = 0.0431 |
|  |  |  |  |  |  | 2 | 55 | [31 66] |  |
| Internal rotation | 1 | 72 | [54 81] | χ^2^ (df) = 5.00 (1)  p = 0.0253 |  |  | | |  |
|  | 2 | 173 | [110 179] |  |  |  |  |  |  |
| External rotation | 1 | 69 | [65 71] | χ^2^ (df) = 0.20 (1)  p = 0.6547 |  |  | | |  |
|  | 2 | 66 | [61 70] |  |  |  |  |  |  |

Condition 1: Scapula posture not adjusted; Condition 2: Scapula posture adjusted;
Ext. rot.: External rotation; Int. rot.: Internal rotation; IQR: Interquartile range; Wilcoxon test: Post-hoc pairwise Wilcoxon signed-rank tests.

Table S2 - Detailed descriptive and inferential statistics for impingement-free ranges of motion

| Motion | Condition | Median  (°) | IQR  (°) | Friedman test | Humeral rotation | Condition | Median (°) | IQR  (°) | Wilcoxon test |
| --- | --- | --- | --- | --- | --- | --- | --- | --- | --- |
| Flexion-Extension | 1 | 159 | [60 198] | χ^2^ (df) = 0.63 (1)  p = 0.4266 | Ext. rot. 60° | 1 | 120 | [31 187] |  |
|  |  |  |  |  |  | 2 | 126 | [94 136] |  |
|  |  |  |  |  | Ext. rot. 30° | 1 | 215 | [164 236] |  |
|  |  |  |  |  |  | 2 | 156 | [152 161] |  |
|  |  |  |  |  | Ext. rot.  0° | 1 | 202 | [187 235] |  |
|  | 2 | 142 | [119 155] |  |  | 2 | 154 | [150 171] |  |
|  |  |  |  |  | Int. rot.  30° | 1 | 135 | [80 185] |  |
|  |  |  |  |  |  | 2 | 142 | [123 149] |  |
|  |  |  |  |  | Int. rot.  60° | 1 | 55 | [29 62] |  |
|  |  |  |  |  |  | 2 | 110 | [94 116] |  |
| Abduction-Adduction | 1 | 114 | [105 124] | χ^2^ (df) = 0.9622 (1)  p = 0.3266 | Ext. rot. 60° | 1 | 124 | [107 128] |  |
|  |  |  |  |  |  | 2 | 66 | [43 85] |  |
|  |  |  |  |  | Ext. rot. 30° | 1 | 123 | [107 128] |  |
|  |  |  |  |  |  | 2 | 111 | [102 127] |  |
|  |  |  |  |  | Ext. rot.  0° | 1 | 115 | [107 129] |  |
|  | 2 | 122 | [98 163] |  |  | 2 | 158 | [116 164] |  |
|  |  |  |  |  | Int. rot.  30° | 1 | 112 | [106 118] |  |
|  |  |  |  |  |  | 2 | 165 | [142 188] |  |
|  |  |  |  |  | Int. rot.  60° | 1 | 103 | [74 111] |  |
|  |  |  |  |  |  | 2 | 162 | [147 177] |  |
| Internal-External rotation | 1 | 142 | [121 145] | χ^2^ (df) = 5.00 (1)  p = 0.0253 |  | | | | |
|  | 2 | 239 | [177 242] |  |  |  |  |  |  |

Condition 1: Scapula posture not adjusted; Condition 2: Scapula posture adjusted;
Ext. rot.: External rotation; Int. rot.: Internal rotation; IQR: Interquartile range; Wilcoxon test: Post-hoc pairwise Wilcoxon signed-rank tests.
