# Supplementary material for: Predicted impingement-free motion amplitudes in reverse total shoulder arthroplasty differs between supine computed tomography and standing biplanar x-ray imaging: a pilot study
Source: JSES Rev Rep Tech. 2025 Jun 14;5(4):1050–7. doi: 10.1016/j.xrrt.2025.05.022 (PMC12573451; doi:10.1016/j.xrrt.2025.05.022)

**Predicted impingement-free motion amplitudes in reverse total shoulder arthroplasty differs between supine CT and standing biplanar X-ray imaging: a pilot study**

Florent Moissenet^1,2^, Sandrine Bousigues^3^, Sana Boudabbous^4^, Davide Cabral^4^, Laurent Gajny^5^, Nicola Hagemeister^6,7^, Nicolas Holzer^2,8^

*^1^ Kinesiology Laboratory, Geneva University Hospitals and University of Geneva, Geneva, Switzerland*

*^2^ Biomechanics Laboratory, Geneva University Hospitals and University of Geneva, Geneva, Switzerland*

*^3^ Laboratoire de Biomécanique et Mécanique des Chocs, Université Gustave Eiffel and Université Claude Bernard Lyon 1, Lyon, France*

*^4^ Department of Radiology, Geneva University Hospitals, Geneva, Switzerland*

*^5^ Arts et Métiers Institute of Technology, Institut de Biomécanique Humaine Georges Charpak, Paris, France*

*^6^ Ecole de Technologie Supérieure, Montréal, Canada*

*^7^ Laboratoire de recherche en imagerie et orthopédie, Centre de recherche du Centre hospitalier de l’Université de Montréal, Montréal, Canada*

*^8^ Orthopaedic Surgery and Musculoskeletal Trauma Care Division, Department of Surgery, Geneva University Hospitals, Geneva, Switzerland*

**Supplementary material 3**

Figure S1 – Orientation of the patient during biplanar X-ray image acquisition (images were taken in 40° axial rotation relative to the anterior-posterior view).


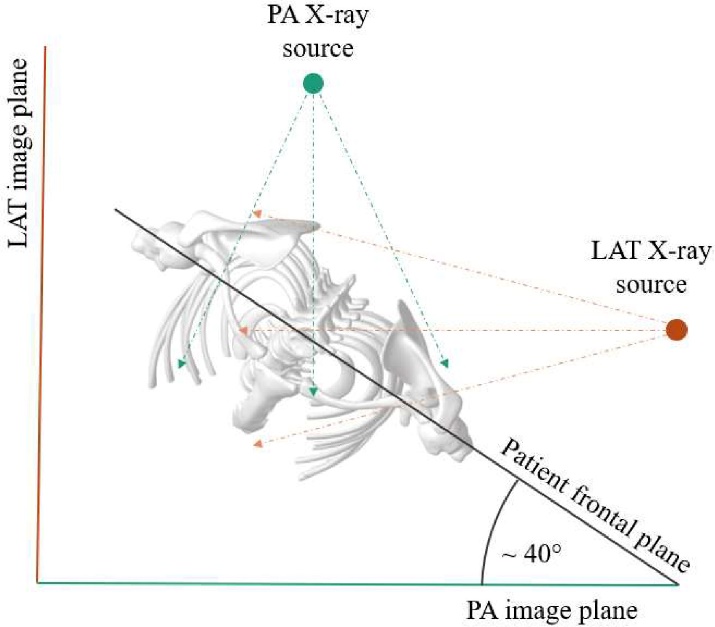

Supplement: Supplementary Material 3 [file mmc3.docx]
